# A strand specific high resolution normalization method for chip-sequencing data employing multiple experimental control measurements

**DOI:** 10.1186/1748-7188-7-2

**Published:** 2012-01-16

**Authors:** Stefan Enroth, Claes R Andersson, Robin Andersson, Claes Wadelius, Mats G Gustafsson, Jan Komorowski

**Affiliations:** 1 The Linnaeus Centre for Bioinformatics, Department of Cell and Molecular Biology, Science for Life Laboratory, Biomedical Center, Uppsala University, Box 598, SE-75124 Uppsala, Sweden; 2 Department of Medical Sciences, Cancer Pharmacology and Computational Medicine, Uppsala University, SE-75185 Uppsala Sweden; 3 Department of Immunology, Genetics and Pathology, Science for Life Laboratory, Rudbeck Laboratory, Uppsala University, SE-75185 Uppsala, Sweden; 4 Interdisciplinary Centre for Mathematical and Computational Modelling, University of Warsaw, PL-02-106 Warszawa, Poland; 5 Department of Immunology, Genetics and Pathology, Science for Life Laboratory, Rudbeck Laboratory, Uppsala University, SE-75185 Uppsala, Sweden; 6 The Bioinformatics Centre, University of Copenhagen, Ole Maaloes Vej 5, DK-2200 Copenhagen N, Denmark

## Abstract

**Background:**

High-throughput sequencing is becoming the standard tool for investigating protein-DNA interactions or epigenetic modifications. However, the data generated will always contain noise due to e.g. repetitive regions or non-specific antibody interactions. The noise will appear in the form of a background distribution of reads that must be taken into account in the downstream analysis, for example when detecting enriched regions (peak-calling). Several reported peak-callers can take experimental measurements of background tag distribution into account when analysing a data set. Unfortunately, the background is only used to adjust peak calling and not as a pre-processing step that aims at discerning the signal from the background noise. A normalization procedure that extracts the signal of interest would be of universal use when investigating genomic patterns.

**Results:**

We formulated such a normalization method based on linear regression and made a proof-of-concept implementation in R and C++. It was tested on simulated as well as on publicly available ChIP-seq data on binding sites for two transcription factors, MAX and FOXA1 and two control samples, Input and IgG. We applied three different peak-callers to (i) raw (un-normalized) data using statistical background models and (ii) raw data with control samples as background and (iii) normalized data without additional control samples as background. The fraction of called regions containing the expected transcription factor binding motif was largest for the normalized data and evaluation with qPCR data for FOXA1 suggested higher sensitivity and specificity using normalized data over raw data with experimental background.

**Conclusions:**

The proposed method can handle several control samples allowing for correction of multiple sources of bias simultaneously. Our evaluation on both synthetic and experimental data suggests that the method is successful in removing background noise.

## Background

High-throughput sequencing of chromatin immunoprecipitated DNA, or ChIP-seq [1], has replaced microarray-based techniques as the standard tool for investigating protein-DNA interactions in the cell. However, the data generated will always contain noise due to sequencing biases, PCR-artefacts, low complexity regions/mappability, chromatin structure or non-specific antibody interactions in the ChIP-step. The noise appears as a background distribution of reads, or tags, which must be taken into account in downstream analyses such as peak-calling.

Experimental assessments of the background read distribution is favoured over purely theoretical and therefore not experimentally validated background models [2]. One such assessment is to sequence the sonicated sample prior to immunoprecipitation (IP). The resulting read distribution is commonly referred to as 'input'. Ideally this distribution would be uniform but Kharchenko *et al *[2] identifies three types of repeatable anomalies that arise in input: singular peaks with very high pile-up, non-uniform wide clusters of increased tag density and, lastly, small clusters of tag densities resembling real peaks but typically with small strand separation where aligned reads pile up in non-meaningful ways. The latter anomaly is difficult to distinguish from a true pile-up. These anomalies are significant to the analysis of ChIP data because the precipitate is a mixture of protein-DNA complexes and bare DNA; ChIP only enriches the protein target and typically only a few percent of the sequenced reads fall within identified peaks [3]. Another source of false positives in ChIP-seq analysis is non-specific binding in the immunoprecipitate. To control for that, the sample can be precipitated using non-specific antiserum, i.e. immunoglobulin G (IgG) that does not have a known antigen in the organism under study. It should be noted that since the degree of enrichment will vary between different antisera an input control experiment adds information to the IgG control. The observed distribution in a specific IP is a mixture of reads due to input anomalies, non-specific and specific IP.

Many of the recently reported peak-callers for ChIP-seq data can make use of control-data to improve predictions of enriched regions. The strategies for correction of background densities vary but are, for instance, performed by simple contrast approaches such as subtraction of the background read distribution from the ChIP-signal or by calculating fold-changes. Other more sophisticated ways of filtering the peaks have been proposed such as using the background read densities as priors in a statistical framework or estimating the false discovery rate. See [2,4] for a discussion of techniques and overview of peak-callers. However, none of the peak-callers offers a way to export the transformed (normalized) raw signal (e.g. ChIP-seq pile-up) actually used for inferring binding sites in the same format as the raw ChIP-seq data. Consequently, there is no way to visualize or compute statistics on the processed signal used internally in the peak-callers to detect enriched regions. The only normalization method published so far seems to be the one introduced by Taslim *et al *[5]. This method yields an output signal with limited resolution due to its use of summary statistics in sliding windows of typically length 1 kb along the genome. This resolution might be sufficient in the application of main interest to Taslim *et al*, which was detection of regions with differential enrichment of RNA polymerase II between conditions, where the exact location of sequenced reads is not required. However, it is an important limitation in applications where fine resolution mappings of for example protein-DNA interactions are studied.

**Figure 1:**
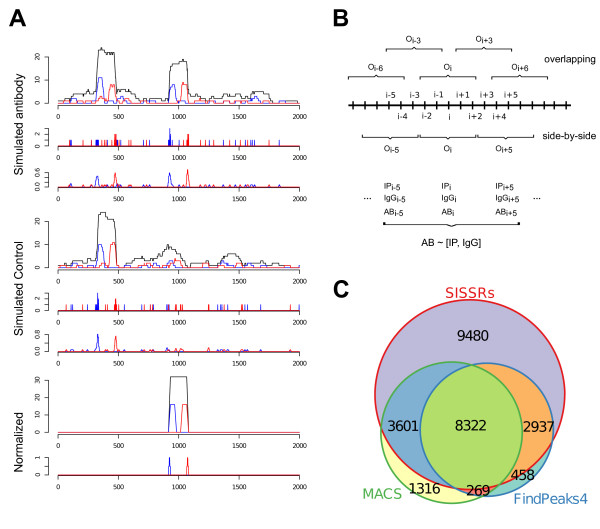
**Strategy overview, synthetic & experimental results**. (A) Results of the proposed normalization strategy on synthetic data. The top three panels represent (from top to bottom): i) the synthetic read signal, (sense in blue, antisense in red and combined in black (XSET)), ii) the individual fragment starts and ends (5' and 3') and iii) the sampled smoothed fragment start-signal used in the normalization step. The next three panels are for a synthetic control signal, and the two bottom panels are the resulting re-created per-bp-signal after normalization. (B) Brief overview of the proposed normalization strategy. For each observation (O_i_) in the genome we calculate a moving window average of the number of start sites in the window centred over *i*, e.g. from i-5 to i+5. The observations, corresponding to the window centres, are taken at intervals that can be shorter than the window size generating 'overlapping' measurements or greater yielding 'side-by-side' windows. The representation of the read counts in the signal used in the proposed normalization procedure is taken as the resulting values for each observation of the centre O_i_. A linear regression fit modelling the AB (antibody) against IP (input) and IGG (IgG) is performed and the residuals are stored. These are finally used to rebuild a per-bp-signal that can be reported in the bed-file format. (C) Number of MAX peaks detected by the three peak finders SISSRs, FindPeaks and MACS using statistical control. The numbers represent peaks found uniquely to the displayed fraction; sizes of the areas reflect the sizes of the sets.

Another issue with ChIP-seq data besides background noise is that different manufactures and versions of sequencing hardware produce reads of different sizes (usually 35-75 nucleotides). To facilitate comparison between different setups it is desirable that the representation of the signal is independent of the read length. There are at least two possibilities to make the representation independent of read-length. One option is to only use counts at the start of aligned reads, the 5' coordinates of reads that aligned the sense strand and the 3' coordinates of reads aligned to the anti-sense strand of that fragment, referred to as 5' and 3' below. Another commonly used option is synthetic *in silico *extension of the read-length to the estimated mean length of the sequenced fragments. The latter results in extended ChIP-seq reads, also known as extended short-read single-end tags (XSETS) [6]. The counts of XSETS can then be added up to produce a combined pile-up signal. Its merit can be seen in Figure 1A where these two different representations of the signal are exemplified. The top panel shows the coverage of reads aligned to the sense (blue) and anti-sense strands (red), and also the combined coverage signal (XSET) in black where each read have been extended with 150 bp. The panels immediately below show the raw (middle) and smoothed (see Methods, bottom) estimates of 5' and 3' locations, respectively. Although there are two clearly visible peaks in the top panel, the 5' and 3' estimates do not surpass three counts at any individual position, which means that it is not easily detected by eye. This is at least partly due to the high biological variation within the cell population and the randomness of the shearing process: the 5' and 3' signals seldom pile up at a single position but rather enrich a small region. The prolonged signal, on the other hand, will generate pile-ups over a larger region, typically centred between the regions enriched by the 5' and 3' signals.

However, synthetically extending sequence reads relies not only on an accurate estimate of the fragment length but also on that the estimate is representative of the distribution of fragments. Hence we focused on a method that produces normalised 5'- and 3'-read counts. Here we present, to our best knowledge, for the first time a normalization algorithm for ChIP-seq data that preserves the high resolution needed to fine map protein-DNA interactions. Since the fragment lengths will vary between experiments we apply an averaging (see Methods) of the 5' and 3' coordinates. The algorithm is based on regression modelling that uses sufficiently small windows (5 bp default) to retain high resolution whilst correcting for one or multiple experimental control measurements simultaneously. We present a demonstration of the strategy on a simulated example data set as well as an in depth evaluation of the normalisation procedure when applied to experimental transcription factor ChIP-sequencing data.

## Results

### Strategy Overview

In order to demonstrate the different components of the strategy, we constructed a small synthetic example data set consisting of a short hypothetical genomic region of 2000 bp (Methods). Our scenario has one binding site (peak) to be inferred, and one added anomaly in input that creates a pile-up of reads that is also observed in IP. The resulting simulated signal was sampled and smoothed (see Methods for details) by averaging over 11 bp (+/- 5 bp) windows every 5 bp, giving an 'overlapping' design as shown in Figure 1B.

The synthetic data and results of the normalization steps are shown in Figure 1A. The top three panels represent (from top to bottom): the synthetic IP signal (sense, anti-sense and combined (XSET)), the read starts (5' and 3') and then the re-sampled smoothed read start signal. This signal is intended to simulate the actual measurement that in practice would be used as input to the normalization procedure. The three panels below are the corresponding results for the synthetic control signals. Finally, the two bottom panels represent the resulting output signal after normalization. Apparently, the only remaining signal after normalization corresponds to the region in the real signal that does not coincide with the peak-region in the control signal. Thus the peak in the real signal that overlapped with the peak in the "control" signal was effectively removed even though the synthetic reads were randomly added in different sized intervals. Note also that the smoothing step effectively removes all the low amplitude noise throughout the region.

### Experimental Data

We evaluated the proposed normalization strategy using two sets of publicly available ChIP-seq data; i) Input, Mouse IgG and a sequence specific transcription factor MAX from the Snyder lab (Yale) [7], obtained for HeLaS3 cells in the ENCODE project [8] and ii) ChIP-seq data set from HepG2 cells consisting of Input data generated within the ENCODE project and a sequence specific transcription factor FOXA1 [9] produced outside of the ENCODE project. The data sets were carefully selected to include only transcription factors with well-characterized DNA-binding motifs. The ENCODE data was sequenced on the Illumina/Solexa platform and the FOXA1 data using a SOLiD instrument from Life Technologies. The Max data for human chromosome 1 was normalized using the two control measurements (Input and Mouse IgG) individually and in combination. The FOXA1 data for human chromosome 1 was normalized using only one control measurement, Input. Note that in this particular experiment the ChIP-seq data and the control data was performed in two different labs using different sequencing platforms. The ENCODE data consisted of 28-32 bp fragments and the FOXA1 data was 50 bp fragments. Normalized signals were generated at the same read length as the ChIP-signals. The resulting normalized data was scanned for peaks using three different peak-callers, i) SISSRs [10], ii) FindPeaks [11] version 4 [12] and finally, iii) MACS [13]. All three peak-callers can either use statistical model as background or generate a specific model based on experimental data. We ran the peak-callers in three ways, i) without experimental background data in which case the statistical modelling was engaged, ii) with experimental background and iii) with normalized data without any additional background. See Methods for full description of parameters in use for each peak-caller. Note that these peak-callers can only benefit from a single control experiment at a time and, consequently, when applicable the comparisons were made normalizing the data using only one control data set at a time. The number of peaks found in any of the datasets employing only statistical control methods, i.e. without use of background control measurements such as IgG or input, was large (Figure 1C) with almost 10,000 peaks specific to the SISSRs peak-finder. In the MAX-signal, SISSRs called over 24,000 peaks on chromosome 1 alone and over 88,000 in Input and IgG, respectively. The other two peak-callers, FindPeaks and MACS, detected over 11,000 and 14,000 peaks respectively in chromosome 1 (Table 1). Note that we do not aim at comparing the performance of the peak-callers to each other, but rather the performance of each peak-caller depending on which background model that was in use or if the input data had been pre-processed by our normalization strategy or not. It should be noted, however, that SISSRs represent an earlier generation of peak-callers than FindPeaks and MACS.

**Table 1:** Number of detected peaks

		SISSRs		findPeaks		MACS	
		
Sample	Control	No. Peaks	% with motif	No. Peaks	% with motif	No. Peaks	% with motif
IgG		88043	1 (1)	6022	1 (2)	2712	2 (2)
Input		93067	3 (4)	9719	5 (5)	10015	3 (4)
MAX		24517	5 (10)	11878	7 (15)	14133	6 (14)
MAX	IgG	3345	12 (30)	4932	10 (26)	18039	6 (12)
MAX	Input	4302	8 (24)	4963	10 (26)	14475	6 (14)
MAX (IgG norm)		1066	26 (51)	3533	16 (36)	2191	13 (38)
MAX (Input norm)		1082	26 (52)	3493	16 (37)	2178	13 (38)
MAX (IgG/Input norm)		1076	26 (53)	3489	16 (37)	2169	13 (38)

Input		13773	2 (2)	47	2 (0)	356	2 (1)
FOXA1		27978	27 (40)	2572	55 (70)	3598	44 (70)
FOXA1	Input	890	58 (71)	2626	57 (72)	3571	42 (70)
FOXA1 (Input norm)		256	73 (75)	599	60 (74)	580	45 (73)

For each data set, the number of peaks detected and the percentages of these that contained the expected binding motif are reported. Numbers within parentheses correspond to the fraction found in the top scoring 20% regions detected.

**Figure 2:**
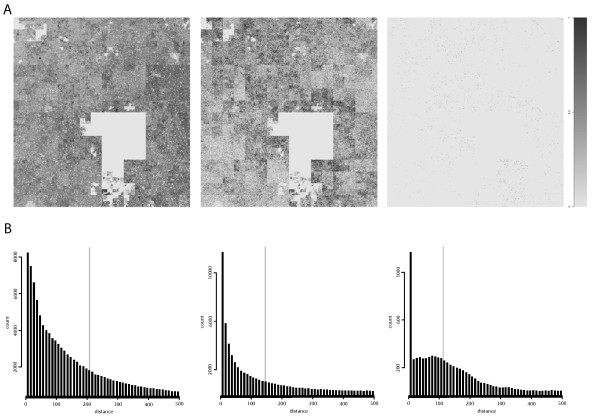
**Properties of chromosome 1**. (A) The aligned fragments for IgG, MAX and MAX normalized with IgG (left to right) are shown for the whole of chromosome 1. The intensities have been normalized to 0.1 within each signal with a darker colour representing more fragments. The large blank part of the figures is the centromeric region of chromosome 1. (B) Estimated distributions of fragment sizes for IgG, MAX and MAX normalized with IgG (left to right), based on distances between sense/anti-sense fragments using the approach taken in Johti *et al *[11]. The plots are produced using the average over 10 bp distance-bins. The grey vertical line indicates the average distance and thus the estimate of the underlying fragment size.

In order to visualize the density of aligned reads we produced so-called Hilbert-curves [14] of the distributions on the entire chromosome 1 for the IgG, MAX and IgG normalised MAX signal (Figure 2A, left to right) and from this it is clear that the IgG-signal is widely distributed over the whole chromosome whilst the MAX-signal seem to be more concentrated. The rightmost panel represent the remaining reads after normalization and this signal is apparently much less abundant than either of IgG or MAX. In addition, we calculated estimates of the lengths of the sequenced fragments as done in Johti *et al *[10] (Figure 2B). In brief, the distribution of reads aligned to the sense and anti-sense are used locally to estimate the length of the sequenced fragments. The fragment size estimate is taken as the average of all such distances in the genome and is depicted as a vertical grey line in the figures. The large occurrence of very short distances in both the IgG and the MAX data is greatly reduced in the normalized data suggesting that the normalized reads more faithfully represent true fragments than the raw signals. The fragment sizes reported by the prime investigator in all these data sets are 200 bp [7].

The number of detected peaks in the normalized data was found to be 1.4 - 6.7 times less for MAX and 3.5 - 6.2 times less for FOXA1 compared to experimental background although with increase percentages of the expected motifs (see below). The latter also holds when investigating a more stringent peak-set consisting of the top 20% of peaks in each category (Table 1) suggesting that the normalization strategy is efficient in reducing false positives among the called peaks.

The use of a sequence specific transcription factors allowed us to estimate the fraction of detected peaks that contained exact matches to the expected binding motif. For MAX this is the E-box, 5'- CACGTG [15] and for FOXA1 5'-TGTTT[AG] [9,13]. Since the peak regions reported vary greatly in length, we used a fixed size for all peaks. For peaks detected using SISSRs, the centre coordinates were prolonged with 75 bp in each direction and the same was done for peaks found with FindPeaks and MACS using the point with the highest score as the centre. The fraction of peaks containing the desired motif for the different data sets is reported in Table 1. Since a very large number of peaks were reported by the peak-finders when no control background data was used, we repeated the analysis using only the top 20% regions in each data set ranked by score reported by the peak-callers. Both analyses resulted in the highest percentages in the peaks called using the normalized data sets.

**Figure 3:**
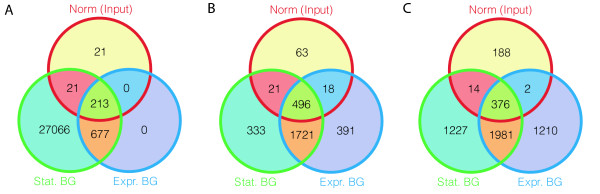
**Comparison of detected peaks for the FOXA1 data**. Overlaps between the peaks called using statistical control (Stat BG), experimental control, Input, (Expr BG) and normalized data (Input) for SISSRs (A), FindPeaks (B) and MACS (C). The numbers represent peaks found uniquely to the displayed fraction; sizes of the areas are not reflecting the sizes of the sets.

We then compared the peaks (regions) found in the two data sets (MAX and FOXA1) by the three peak finders under three different conditions; (i) using the data with statistical background, (ii) using the data plus one experimental control data set, Input, and finally (iii) using the normalized data against the same control data as in (ii). The number of regions overlapping by at least 1 bp for the FOXA1 results is depicted in Figure 3A-C. Overall, in the sets containing overlaps with the results using normalized data, the percentage of peaks containing the expected motifs is higher. In particular, this is true for the called peaks common between the statistical background and the Input-normalized data but not detected using experimental background compared to peaks common between statistical and experimental background not detected in the normalized data. For MAX, 15.7-25.0% of the former regions contained the expected motif and 6.0-9.7% for the latter. For FOXA1 these numbers were 66.9-70.5% compared to 48.6 - 58.9%. The presence of such regions found using the normalized data indicates that there are regions in the data that would otherwise have been missed by the peak-caller when using experimental background, and that these regions contained a high fraction of the expected motif, suggesting that they are indeed true positives. For the MAX-data, we also compared the peaks detected using either or both of the control measurements in the normalization (Table 1). For this data, we found very little difference in the regions detected suggesting that, in this case, IgG and input performed similarly as control experiments. Lastly, we find that on the normalized data, the results from using the earlier generation peak-caller SISSRs is quite comparable to the results obtained by the other peak-callers.

**Figure 4:**
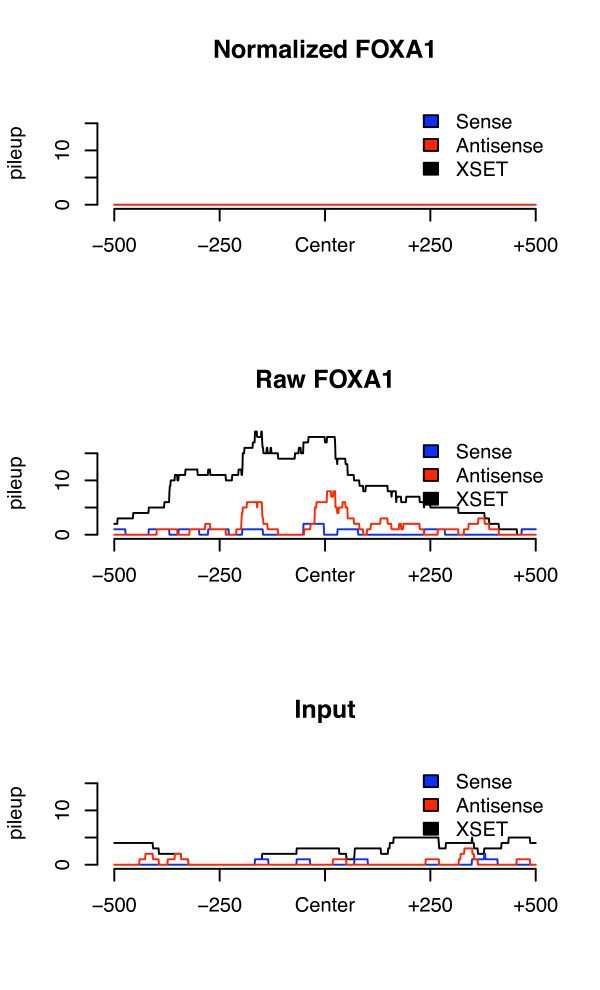
**Results of the normalization strategy on experimental data**. Normalized, raw and Input signals are shown over the top-ranked peak unique to the analysis of FOXA1 data in HepG2 using Input as background to the FindPeaks peak caller. The region covered by the peak does not contain the expected motif.

It is interesting that, when using only the top scoring peaks from each peak-caller, the results, in terms of motif containing regions, are fairly equal regardless of the type of data used: statistical background, experimental background or normalized data. This is especially evident for the lower scoring MAX data. This indicates that the regions removed by the normalization method contain a high fraction of false positives. We also calculated the fraction of peaks containing the desired motif for the set of peaks that was either unique to experimental vs. statistical background but overlapped with the normalized data and the general conclusion is that the subsets that overlap with peak-regions detected using the normalized data contains a higher fraction of peaks with the desired motif than a subset that does not have regions in common with the normalized data. This also suggests a higher fraction of true positives. An example of a peak not discovered in the normalized data but ranked among the top peaks unique to the analysis with experimental background for FOXA1 is shown in Figure 4.

For the FOXA1 data, the original investigator [9] performed 22 qPCR validations of 15 positive regions and 7 negative. We extracted and normalized the ChIP and Input signal +/-250 kb around these sites and ran SISSRs and findPeaks on the raw (statistical and experimental background) and on the normalized data (Additional file 1, Figure S1). The performance in terms of sensitivity and specificity on this small sample set (n = 22) was higher for the normalized data for both peak-callers (0.87/0.57 and 0.67/0.86 for findPeaks and SISSRs respectively) compared to the second best (experimental background) performance (0/1 and 0.60/0.43).

## Conclusions

Normalizing is a vital part of any next generation sequencing study. For microarray based techniques there exist many different types of normalizing methods directed at different sources of bias (e.g. dye effects or background noise). To the best of our knowledge, up until now, there has not been any normalization method for ChIP-sequencing data that globally addresses effects, such as non-specific antibody interactions or background noise, which can be suppressed using control experiments. Many of the existing peak-callers are tailor-made for ChIP-sequencing data and can make use of a background model based on experimental control data, rather than purely theoretical statistical assumptions, to filter out regions that are also enriched in the control data. However, these approaches are inherently designed to be used for peak calling and are therefore not easily transformed into universal normalization methods. In order to be fully compliant with any type of analysis performed on ChIP-seq data it is also imperative that the resulting normalized signal is reported in the same format as the raw ChIP-seq data.

Here we present for the first time such a universal normalization strategy based on a simple regression framework. The resulting method does not destroy the fine resolution obtained in next generation sequencing data and relies on re-sampling of the fragment starting points in small intervals, typically 5 base pairs long. At least for the data examined here this gave a reasonable trade-off between keeping the high resolution and the underlying biological variance between samples. Since the linear regression modelling used by this new method may be fitted using standard software libraries for ordinary least squares regression, it is very easy to include in any software library for analysis of ChIP-seq data.

Finally, the options to include more than one control data sets allows an investigator to for instance account for technical error sources such as unspecific interactions of the antibody and for biologically less likely active sites as defined by e.g. nucleosome occupancy or any histone modification data set.

## Methods

### Data acquisition

The ENCODE data sets where downloaded from the repositories using the UCSC genome browser [16]. The FOXA1 data was collected from a previous in-house project and is available from the European Nucleotide Archive under accession number ERP000005.

### Synthetic Data Generation

The synthetic data was generated in a small hypothetical region of 2000 bp containing two peaks in the ChIP-signal one of which had a similar peak in the Control data. The pile-ups were generated by placing reads at random inside pre-defined short intervals (peaks). The endpoints of the intervals are intended to represent the extreme borders of sonicated fragments, and sense (5') and anti-sense (3') reads were placed within the intervals corresponding to the start and end of the sonicated fragment respectively. The script for generating the simulated data is included in the Additional files. The interval lengths were empirically set to 20 bp for the synthetic IP data and to 25 for the synthetic control in order to simulate less variation in the IP data compared to the control. In total 40 reads were assigned to the real signal and 25 to the control in this 2000 bp region. In addition, 20 noise fragments were added on each strand at positions drawn at random from a uniform distribution over the whole region.

### Algorithm

The underlying model of our normalization method assumes that the measured raw signal may be accurately described as a linear combination of three following components: (i) the *de facto *interaction sites of the investigated protein, (ii) non-specific anti-body interaction and (iii) background resulting from sequencing biases, low complexity regions/mappability, chromatin structure of other so far uncharacterized effects. An input-measurement, which is taken from the chromatin sample before any antibody pull down is done, is assumed not to be specifically enriched for any of the three components listed above. Both the non-specific and the specific antibody experiments are assumed to be enriched for components (i) and (ii) compared to (iii).

In the method proposed here, including both IgG and input as controls, the linear model for the observed ChIP-seq signal can be written as

(1) $${\tilde{t}}_{i}=\alpha t_{i}+\beta b_{i}+\gamma g_{i}+e^{\prime }$$

Here the subscript *i *denotes position, ${\tilde{t}}_{i}$ is the observed signal, *t_i _*the signal arising from specific antibody binding, *b_i _*background, *g_i _*the signal due to non-specific binding of the antibody and *e' *will throughout denote noise that is orthogonal to *t_i_*. Thus α, β and γ are coefficients in the linear model and we assume that the noise is additive and that *t *is uncorrelated with (orthogonal to) *b *and *g*. In addition to the observed signal ${\tilde{t}}_{i}$ we assume the experimenter has access to observed background

(2) $${\tilde{b}}_{i}=b_{i}+e^{\prime \prime }$$

and IgG signal

(3) $${\tilde{g}}_{i}=\beta^{\prime }b_{i}+\gamma^{\prime }g_{i}+e^{\prime \prime \prime }.$$

The case when only (2) or (3) is available follows simply from the description below.

The underlying objective of the normalization procedure proposed herein is to obtain a scaled estimate of the true signal *t_i _*(caused by antibody binding). From (1) it follows that an estimate ${\hat{t}}_{i}$of *αt_i _*can be obtained as ${\hat{t}}_{i}={\tilde{t}}_{i}-{\hat{n}}_{i}$, where ${\hat{n}}_{i}$ estimates *n_i _*= *βb_i _*+ *γg_i _*+ *e'*. Now, consider the sum of squares

(4) $$\sum_{}{\left({\tilde{t}}_{i}-{\hat{n}}_{i}\right)}^{2}=\sum_{i}{\left(\alpha t_{i}+n_{i}-{\hat{n}}_{i}\right)}^{2}=\alpha^{2}\sum_{i}t_{i}^{2}+2\alpha \sum_{i}t_{i}(n_{i}-{\hat{n}}_{i})+\sum_{i}{\left(n_{i}-{\hat{n}}_{i}\right)}^{2}.$$

By assumption $\underset{i}{\sum }t_{i}n_{i}=0$ so choosing the estimate to minimize $\sum_{i}{\left({\tilde{t}}_{i}-{\hat{n}}_{i}\right)}^{2}$ will also minimize

(5) $$\underset{i}{\sum }{\left(n_{i}-{\hat{n}}_{i}\right)}^{2}-2\alpha \underset{i}{\sum }t_{i}{\hat{n}}_{i}.$$

As the estimator we use

(6) $${\hat{n}}_{i}=\hat{u}{\tilde{b}}_{i}+\hat{v}{\tilde{g}}_{i}.$$

Inserting (2) and (3) into (6) shows that $\sum_{i}t_{i}{\hat{n}}_{i}=0$ and thus

(7) $$\text{arg}\;\underset{\hat{u},\hat{v}}{\text{min}}\underset{i}{\sum }{\left(n_{i}-{\hat{n}}_{i}\right)}^{2}=\text{arg}\;\underset{\hat{u},\hat{v}}{\text{min}}\underset{i}{\sum }{\left({\tilde{t}}_{i}-{\hat{n}}_{i}\right)}^{2}$$

Consequently, the least squares estimate for *n_i _*is obtained for the least squares estimate for ${\tilde{t}}_{i}$. The values *û* and $\hat{v}$ that minimize (7) is the ordinary least squares regression solution when predicting ${\tilde{t}}_{i}$ from ${\tilde{b}}_{i}$ and${\tilde{g}}_{i}$. Moreover the estimate of *αt_i _*in each position *i *is obtained as the residual of the regression for that positions value, i.e. ${\hat{t}}_{i}={\tilde{t}}_{i}-{\hat{n}}_{i}$ and the estimates are easily calculated using any software library that offers least squares regression modelling. Furthermore, we note that the methodology can be extended to use more than two control experiments and that the basic idea of removing uncorrelated noise from a signal by using measurements of sources correlated to the noise has previously been applied in adaptive noise cancelling [17].

In our implementation, the normalization is done locally, in sections of 100 k. The section size is basically limited by the memory capacity of the system. In our experience, however, the section size does not generally affect the results (Additional file 1, Table S1). This allows for local usage of the algorithm in specific subsections of a genome and therefore simultaneous (parallel) processing of different regions. If a promoter specific transcription factor is investigated the normalization can be applied to promoter regions alone, reducing computational time. In each section, all analyzed signals (e.g. ChIP-data and controls) are smoothed reporting the mean over small windows (typically +/- 5 bp). This smoothed signal is then sampled at given intervals (typically 5 bp) that in fact serve as a size-reduction step where we only retain the information of the centre position of the averaging window. The smoothed and sampled signals (ChIP and controls) are then used as starting point for the regression. Note that the average-windows can overlap depending on how the window size and centre-to-centre distance is chosen. In such case, the read count on a given base pair will contribute to several windows contributing to an even smoother signal. After the normalization a per-bp-signal output signal can be rebuilt from the centre-averages filling in missing values between centres with e.g. the value of the centres or the averages between adjacent centres. Since the analysis is split on read aligning to the sense or anti-sense strand, the resulting per-bp-signal can easily be written out in the same format as aligned reads, retaining the strand specificity. Here we have chosen to work with the BED-format and write out dummy reads with the same length as the original sequenced reads. The major steps of the algorithm are outlined in Figure 1A. A major strength of this new normalization method is that there is not really any need to account for different sequencing depths in the different signals as this is handled by the models created in the regression step. Specifically, differences in sequencing depths will reflect as different scaling of the coefficients of the regression model and any prior scaling of the signals will only amount to other, scaled, coefficients.

### Implementation

For demonstration purposes we implemented the algorithm using R [18]. In order to be compliant with the already established downstream analysis (peak finders) the program outputs dummy reads in the BED-format located at all positions with a residual after regression greater than 1. The number of such dummy reads at each position was taken as the largest whole number portion of the residual at that position. The R-source code needed to reproduce the low level analysis of this work is available in Additional file 2. The R-script requires R version 2.10 or higher, additional packages and software [10,11,18,19]. The generation of the Hilbert-curves required a 64-bit system with proper version of R and additional packages. The overlaps between regions and extraction of sequences were done using BEDTools [19]. Signal footprints were produced using the SICTIN [20] software suite. The algorithm has also been implemented as a command line program in C++ using the GNU Scientific Library [21] for performing the regression. The source code is publicly available at https://github.com/ project name "Strand-Specific-Normalization-of-ChIP-seq-Data".

### Peakfinders

The three peak-finders used here were SISSRs (version 1.4), FindPeaks 4.0 (version 4.0.15) and MACS (version 1.4.0rc2). The peak-finders where run with the following parameters in effect (only non-default settings are reported here):

SISSRs, "-s 3093120360". FindPeaks, statistical background, "-dist_type 0 < fraglength as reported > -subpeaks 0.5 -landerwaterman 0.001". FindPeaks, experimental background, "-control < file > -dist_type 1 < fraglength as reported > -subpeaks 0.5". MACS, " -g hs --bw < fraglength as reported > --shiftsize < fraglength as reported > --call-subpeaks --wig"

## Competing interests

The authors declare that they have no competing interests.

## Authors' contributions

SE and CRA conceived of the study, its design and wrote the manuscript. MGG, CW and JK participated in the design of the study and manuscript writing. RA participated in the design of the study. All authors read and approved the final manuscript.

## Supplementary Material

Additional file 1 **Supplementary Data**. One additional figure and one table. Click here for file

Additional file 2 **rscript.R**. The implementation of the algorithm in R and some code used to download raw data and tools. Click here for file

## References

[B1] Johnson DS Mortazavi A Myers RM Wold B Genome-wide mapping of in vivo protein-DNA interactions Science 2007 316 1497 1502 10.1126/science.1141319 17540862

[B2] Kharchenko PV Tolstorukov MY Park PJ Design and analysis of ChIP-seq experiments for DNA-binding proteins Nat Biotechnol 2008 26 1351 1359 10.1038/nbt.1508 19029915 PMC2597701

[B3] Hoffman BG Jones SJ Genome-wide identification of DNA-protein interactions using chromatin immunoprecipitation coupled with flow cell sequencing J Endocrinol 2009 201 1 13 10.1677/JOE-08-0526 19136617

[B4] Laajala TD Raghav S Tuomela S Lahesmaa R Aittokallio T Elo LL A practical comparison of methods for detecting transcription factor binding sites in ChIP-seq experiments BMC Genomics 2009 10 618 10.1186/1471-2164-10-618 20017957 PMC2804666

[B5] Taslim C Wu J Yan P Singer G Parvin J Huang T Lin S Huang K Comparative study on ChIP-seq data: normalization and binding pattern characterization Bioinformatics 2009 25 2334 2340 10.1093/bioinformatics/btp384 19561022 PMC2800347

[B6] Robertson G Hirst M Bainbridge M Bilenky M Zhao Y Zeng T Euskirchen G Bernier B Varhol R Delaney A Genome-wide profiles of STAT1 DNA association using chromatin immunoprecipitation and massively parallel sequencing Nat Methods 2007 4 651 657 10.1038/nmeth1068 17558387

[B7] ENCODE Data Coordination Center at UCSC, Yale data http://hgdownload.cse.ucsc.edu/goldenPath/hg18/encodeDCC/wgEncodeYaleChIPseq/

[B8] Birney E Stamatoyannopoulos JA Dutta A Guigo R Gingeras TR Margulies EH Weng Z Snyder M Dermitzakis ET Thurman RE Identification and analysis of functional elements in 1% of the human genome by the ENCODE pilot project Nature 2007 447 799 816 10.1038/nature05874 17571346 PMC2212820

[B9] Motallebipour M Ameur A Reddy Bysani MS Patra K Wallerman O Mangion J Barker MA McKernan KJ Komorowski J Wadelius C Differential binding and co-binding pattern of FOXA1 and FOXA3 and their relation to H3K4me3 in HepG2 cells revealed by ChIP-seq Genome Biol 2009 10 R129 10.1186/gb-2009-10-11-r129 19919681 PMC3091322

[B10] Jothi R Cuddapah S Barski A Cui K Zhao K Genome-wide identification of in vivo protein-DNA binding sites from ChIP-Seq data Nucleic Acids Res 2008 36 5221 5231 10.1093/nar/gkn488 18684996 PMC2532738

[B11] Fejes AP Robertson G Bilenky M Varhol R Bainbridge M Jones SJ FindPeaks 3.1: a tool for identifying areas of enrichment from massively parallel short-read sequencing technology Bioinformatics 2008 24 1729 1730 10.1093/bioinformatics/btn305 18599518 PMC2638869

[B12] Findpeaks 4.0 http://sourceforge.net/apps/mediawiki/vancouvershortr/index.php?title=FindPeaks#FindPeaks_4.0

[B13] Zhang Y Liu T Meyer CA Eeckhoute J Johnson DS Bernstein BE Nusbaum C Myers RM Brown M Li W Liu XS Model-based analysis of ChIP-Seq (MACS) Genome Biol 2008 9 R137 10.1186/gb-2008-9-9-r137 18798982 PMC2592715

[B14] Anders S Visualization of genomic data with the Hilbert curve Bioinformatics 2009 25 1231 1235 10.1093/bioinformatics/btp152 19297348 PMC2677744

[B15] Luscher B Function and regulation of the transcription factors of the Myc/Max/Mad network Gene 2001 277 1 14 10.1016/S0378-1119(01)00697-7 11602341

[B16] Rosenbloom KR Dreszer TR Pheasant M Barber GP Meyer LR Pohl A Raney BJ Wang T Hinrichs AS Zweig AS ENCODE whole-genome data in the UCSC Genome Browser Nucleic Acids Res 2010 38 D620 625 10.1093/nar/gkp961 19920125 PMC2808953

[B17] Widrow B Glover JR McCool JM Kaunitz J Williams CS Hearn RH Zeidler JR Dong E Goodlin RC ADAPTIVE NOISE CANCELLING - PRINCIPLES AND APPLICATIONS Proc IEEE 1975 63 1692 1716

[B18] R Development Core Team. R: A language and environment for statistical computing. R Foundation for Statistical Computing, Vienna, Austria. ISBN 3-900051-07-0 2009 http://www.R-project.org

[B19] Quinlan AR Hall IM BEDTools: a flexible suite of utilities for comparing genomic features Bioinformatics 2010 26 841 842 10.1093/bioinformatics/btq033 20110278 PMC2832824

[B20] Enroth S Andersson R Wadelius C Komorowski J SICTIN: Rapid footprinting of massively parallel sequencing data BioData Min 2010 3 4 10.1186/1756-0381-3-4 20707885 PMC2928217

[B21] M Galassi JD Theiler J Gough B Jungman G Alken P Booth M Rossi F GNU Scientific Library Reference Manual 3

