# Supplementary material for: A strand specific high resolution normalization method for chip-sequencing data employing multiple experimental control measurements
Source: Algorithms Mol Biol. 2012 Jan 16;7:2. doi: 10.1186/1748-7188-7-2 (PMC3371767; doi:10.1186/1748-7188-7-2)
Supplement: Additional file 1 — Supplementary Data. One additional figure and one table. [file 1748-7188-7-2-S1.DOC]

# Supplementary Data

# A strand specific high resolution normalization method for chip-sequencing data employing multiple experimental control measurements

### S. Enroth^†^, C. R. Andersson^†^, R. Andersson, C. Wadelius, M.G. Gustafsson and J. Komorowski

**
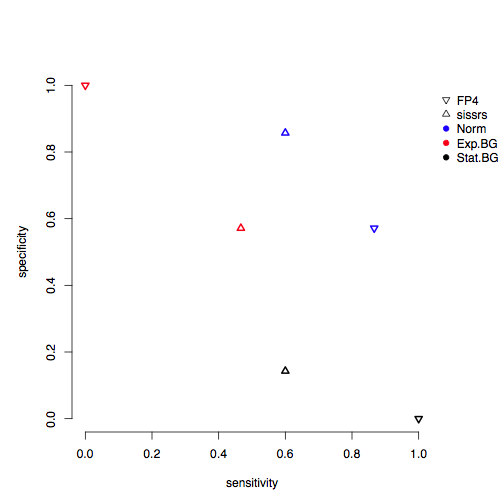
**

**Supplementary Figure 1:** Sensitivity and Specificity estimations. 22 (15 positive and 7 negative) qPCR validated FOXA1 interaction sites was used to estimate sensitivity and specificity. The qPCR results were taken from Motallebipour et al [1]. Data from +/-100kb around the qPCR region was collected and normalized using Input. Peak-finders were then run on i) Input-normalized data (Norm), ii) Raw data using Input as background (Exp.BG) and iii) Raw data with a statistical background only (Stat. BG). True positives (TP) were defined if at least 1bp of the called peaks (regions) overlapped with a positive qPCR region and True negative (TN) where no region overlapped in any part with a negative qPCR region. False Positives (FP) were defined when a called peak overlapped at least 1 bp with any negative qPCR region and False Negatives (FN) when no peaks where called that overlapped a positive region with at least 1 bp. Overlaps were calculated using BEDTools[2].

|  |  | Percentage of common non-zero positions | | | |
| --- | --- | --- | --- | --- | --- |
|  | Chunk size | 25k | 50k | 100k | 200k |
| ‘+’ Strand |  |  |  |  |  |
| Pearsons’ R^2^ | 25k |  | 99.59 % | 99.89 % | 99.84 % |
|  | 50k | 0.99989 |  | 99.94 % | 99.89 % |
|  | 100k | 0.99989 | 1 |  | 99.95 % |
|  | 200k | 0.99989 | 1 | 0.99994 |  |
| ‘-‘ Strand |  |  |  |  |  |
| Pearsons’ R^2^ | 25k |  | 99.94 % | 99.91 % | 99.61 % |
|  | 50k | 0.99995 |  | 99.98 % | 99.98 % |
|  | 100k | 0.99978 | 0.99982 |  | 99.70 % |
|  | 200k | 0.99977 | 0.99975 | 0.99993 |  |

**Supplementary Table 1:** Effects of chunk size on normalized data split on strand. Upper-right part reports common non-zero positions as percentage of all non-zero positions. Lower-left part reports Pearsons’s R^2^ between values from different chunk sizes over common non-zero positions.

1. Motallebipour M, Ameur A, Reddy Bysani MS, Patra K, Wallerman O, Mangion J, Barker MA, McKernan KJ, Komorowski J, Wadelius C: **Differential binding and co-binding pattern of FOXA1 and FOXA3 and their relation to H3K4me3 in HepG2 cells revealed by ChIP-seq.** *Genome Biol* 2009, **10:**R129.

2. Quinlan AR, Hall IM: **BEDTools: a flexible suite of utilities for comparing genomic features.** *Bioinformatics* 2010, **26:**841-842.
